# A homozygous nonsense variant in the alternatively spliced *VLDLR* exon 4 causes a neurodevelopmental disorder without features of *VLDLR* cerebellar hypoplasia

**DOI:** 10.1038/s10038-024-01279-w

**Published:** 2024-07-31

**Authors:** Tess Holling, Ibrahim M. Abdelrazek, Ghada M. Elhady, Marwa Abd Elmaksoud, Seung Woo Ryu, Ebtesam Abdalla, Kerstin Kutsche

**Affiliations:** 1 Institute of Human Genetics, University Medical Center Hamburg-Eppendorf, Hamburg, Germany; 2 Department of Human Genetics, Medical Research Institute, Alexandria University, Alexandria, Egypt; 3 Neurology Unit, Pediatric Department, Faculty of Medicine, Alexandria University, Alexandria, Egypt; 4 3billion Inc., Seoul, South Korea

**Keywords:** Genetics research, Neurological disorders

## Abstract

*VLDLR* cerebellar hypoplasia is characterized by intellectual disability, non-progressive cerebellar ataxia, and seizures. The characteristic MRI findings include hypoplasia of the inferior portion of the cerebellar vermis and hemispheres, simplified cortical gyration, and a small brain stem. Biallelic *VLDLR* pathogenic variants cause loss-of-function of the encoded very low-density lipoprotein receptor. *VLDLR* exons 4 and 16 are alternatively spliced, resulting in the expression of four transcript variants, including two exon 4-lacking mRNAs expressed in the human brain. Previously reported *VLDLR* pathogenic variants affect all four transcript variants. Here we report on two sisters with facial dysmorphism, microcephaly, intellectual disability, and normal brain imaging. Exome sequencing in one patient identified the homozygous *VLDLR* nonsense variant c.376C>T; p.(Gln126*) in exon 4; her similarly affected sister also carried the homozygous variant and parents were heterozygous carriers. *VLDLR* transcript analysis identified mRNAs with and without exon 4 in patient fibroblasts, while exon 4-containing *VLDLR* mRNAs were predominantly detected in control fibroblasts. We found significantly reduced *VLDLR* mRNA levels in patient compared to control cells, likely caused by nonsense-mediated mRNA decay of exon 4-containing *VLDLR* transcripts. Expression of neuronal VLDLR isoforms produced from exon 4-lacking transcripts may have protected both patients from developing the cerebellar hypoplasia phenotype.

## Introduction

Biallelic pathogenic variants in the *VLDLR* gene (MIM 192977) cause *VLDLR* cerebellar hypoplasia, which is a subtype of dysequilibrium syndrome that combines non-progressive cerebellar ataxia with moderate to profound intellectual disability (ID) (MIM 224050). Affected individuals have truncal ataxia, which leads to non-ambulation, quadrupedal ambulation, or achievement of bipedal ambulation in late childhood. Dysarthria and strabismus are common. Brain MRIs reveal a typical brain malformation pattern consisting of hypoplasia of the inferior portion of the cerebellar vermis and hemispheres, simplified gyration of the cortex, and a small brain stem. Facultative anomalies are epileptic seizures, microcephaly, and short stature [1, 2].

**Fig. 1:**
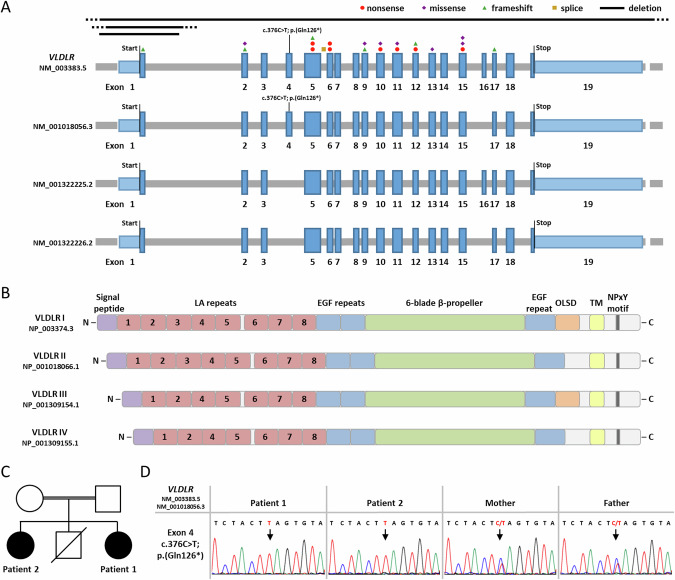
VLDLR transcript variants and isoforms with published and novel *VLDLR* pathogenic variants, family pedigree, and Sanger sequence traces showing the *VLDLR* nonsense variant c.376C > T. **A** Schematics of the exon-intron structure and transcript variants of the *VLDLR* gene listed at NCBI (last accessed 03/2024: NM_003383.5, NM_001018056.3, NM_001322225.2, and NM_001322226.2). Exons are indicated by boxes and introns by gray lines. Untranslated regions are depicted in light and the coding region in dark blue. Start and stop codons are indicated. The newly identified nonsense variant c.376C > T; p.(Gln126*) is located in exon 4. The location of published *VLDLR* pathogenic variants is shown: nonsense variants (red circles), missense variants (violet diamonds), frameshift variants (green triangles), splice site variant (yellow square), and exon-spanning deletions (black lines) (Supplementary Table S1). **B** Domain structure of the four VLDLR isoforms according to UniProtKB (isoform I: NP_003374.3; isoform II: NP_001018066.1; isoform III: NP_001309154.1; isoform IV: NP_001309155.1). Colored boxes represent the indicated domains (violet: signal peptide; red: LDL receptor type A (LA) repeats; blue: EGF repeat; green: 6-blade β-propeller; orange: *O*-linked sugar domain (OLSD); yellow: transmembrane domain (TM); dark grey: NPxY motif). **C** Pedigree of the family. The two affected sisters, patients 1 and 2, are indicated by a black symbol. A brother died at the age of 8 months of unknown cause. **D** Sequence traces show the *VLDLR* nonsense variant c.376C > T; p.(Gln126*) in leukocyte-derived DNA from patients 1 and 2 in the homozygous and in mother and father in the heterozygous state. An arrow points to the nucleotide change. N: N-terminus; C: C-terminus

To date, more than 70 individuals have been reported with homozygous or compound heterozygous *VLDLR* variants (Supplementary Table S1). The molecular genetic spectrum encompasses nonsense, frameshift, missense, and splice site variants as well as large deletions. The variants are scattered across the gene and affect 11 of the 19 exons (Fig. 1A). The previously reported *VLDLR* pathogenic variants are predicted to result in partial or complete loss of function of the VLDLR protein [2].

*VLDLR* encodes the very low-density lipoprotein receptor, which consists of eight LDL receptor type A repeats (LA repeats) in the N-terminus followed by two epidermal growth factor precursor-like repeats, a YWTD β-propeller, an *O*-linked sugar domain (OLSD), a transmembrane domain, and a C-terminal cytoplasmic domain [3, 4]. Alternative splicing of *VLDLR* exons 4 and 16 leads to expression of four different transcript variants in human brain (NM_003383.5, NM_001018056.3, NM_001322225.2, and NM_001322226.2) (Fig. 1A): one encodes the full-length VLDLR-I protein, the transcript without exon 16 codes for VLDLR-II lacking the OLSD, the mRNA without exon 4 encodes VLDLR-III lacking the third LA repeat, and the fourth transcript lacks exons 4 and 16 (Fig. 1B) [5–7].

Here we report on two sisters with microcephaly, intellectual disability, and normal brain imaging who carried the homozygous nonsense variant c.376C > T; p.(Gln126*) in the alternatively spliced exon 4 of *VLDLR*. Data from our *VLDLR* transcript analysis in patient fibroblasts together with literature data suggest that brain expression of *VLDLR* transcripts without exon 4 encoding VLDLR isoforms III and IV may have protected both siblings from developing the more severe phenotype.

## Material and Methods

### Whole-exome sequencing and variant calling

Genomic DNA was extracted from leukocytes of patients 1 and 2 and their parents by standard procedures. Whole-exome sequencing (WES) was performed on genomic DNA of patient 1 by 3billion (Seoul, Republic of Korea). Exome capture was performed using the xGen Exome Research Panel v2 (Integrated DNA Technologies, Coralville, Iowa, USA) and sequencing was performed using NovaSeq 6000 (Illumina, San Diego, CA, USA). In total, 8,833,672,144 bases of sequence were generated and uniquely aligned to the Genome Reference Consortium Human Build 37 (GRCh37) and Revised Cambridge Reference Sequence (rCRS) of the mitochondrial genome, generating 130.34 mean depth-of-coverage within the 34,366,188 bases of the captured region, which is approximately 99.3% of the RefSeq protein coding region. Approximately 98.80% of the targeted bases were covered to a depth of ≥20x. Gene or exon-level depth-of-coverage information is available upon request. In total, 67,013 single nucleotide variants (SNV) and 10,747 small insertions and deletions (indel) were identified. The variant interpretation was performed using EVIDENCE, a software developed in-house (3 billion) to prioritize variants based on the guidelines recommended by the American College of Medical Genetics and Genomics (ACMG) and the Association for Molecular Pathology (AMP) [8] in the context of the patient’s phenotype, relevant family history and previous test results provided by the ordering physician [9].

### Variant validation by Sanger sequencing

Sanger sequencing permitted *VLDLR* variant validation and/or segregation in leukocyte- and/or fibroblast-derived DNA from patients 1 and 2 and parents. Primer sequences are in Supplementary Table S2. PCR amplicons were directly sequenced using the ABI BigDye Terminator Sequencing kit (Applied Biosystems) and an automated capillary sequencer (ABI 3500, Applied Biosystems). Sequence electropherograms were analyzed using SeqManPro™ (DNASTAR® Software for Life Scientists) and Chromas Lite 2.1.1 (Technelysium Pty Ltd). The *VLDLR* variants were described according to the GenBank reference sequences NM_003383.5 and NP_003374.3. Correct variant nomenclature was assessed using Mutalyzer (https://mutalyzer.nl/name-checker). The *VLDLR* variant c.376C> T; p.(Gln126*) was submitted to the LOVD database (https://databases.lovd.nl/shared/genes/VLDLR), with LOVD Variant IDs # 0000972221 and #0000972222.

### Cell culture

Primary fibroblasts obtained from skin biopsies of patient 1, patient 2, and four healthy individuals (control 1: 4-year-old female; control 2: 4-year-old female; control 3: 16-year-old male; control 4: 9-year-old male) were cultured in Dulbecco’s modified Eagle’s medium (DMEM; Thermo Fisher Scientific) supplemented with 10% FBS (GE Healthcare) and penicillin-streptomycin (100 U/mL and 100 µg/mL, respectively; Thermo Fisher Scientific) and incubated at 37 °C in a humidified atmosphere with 5% CO_2_. Cells were tested for mycoplasma contamination by PCR and were confirmed to be mycoplasma-free.

### RNA isolation, cDNA synthesis, and qualitative and quantitative transcript analysis

Total RNA was extracted from patient-derived and control fibroblasts (Monarch Total RNA Miniprep Kit, New England Biolabs). RNA concentration and purity of the samples were assessed by use of the Epoch™ Microplate Spectrophotometer (BioTek). Total RNA from human brain cerebellum (Zyagen, #HR-202) and human fetal brain (BioChain®, #R1244035-50) were purchased. Total RNA was reverse transcribed into cDNA according to the manufacturer’s instructions (LunaScript® RT SuperMix Kit, New England Biolabs).

RT-PCR amplicons were generated according to standard PCR protocols with the OneTaq® Quick-Load 2 × Master Mix (New England Biolabs). Primer sequences are in Supplementary Table S2. Selected RT-PCR products were either directly Sanger-sequenced (see above) or cloned into the pCR2.1 TOPO TA Cloning Vector (Thermo Fisher Scientific) according to the manufacturer’s instructions. Individual *Escherichia coli* clones were subjected to colony PCR and PCR products were directly Sanger-sequenced (see above).

The SYBR Green I-based Luna Universal qPCR Master Mix (New England BioLabs) was used for the relative quantification of *VLDLR* mRNA levels by real-time quantitative PCR (RT-qPCR). Primer sequences are in Supplementary Table S2. Technical triplicates of RT-qPCR samples were prepared as a 10 µL approach. The PCR conditions included initial denaturation at 95 °C for 5 min, followed by 40 cycles of 30 s at 95 °C, 30 s at 58 °C and 45 s at 72 °C. PCR amplification specificity was determined by melting curve analysis with a range from 60 °C to 95 °C. The RT-qPCR reaction was performed using the QuantStudio 3 Real-Time PCR System (Thermo Fisher Scientific). The values of the cycle threshold (CT) of *VLDLR* transcripts were normalized to transcripts of the housekeeping gene *GAPDH*. For relative gene expression, the comparative cycle threshold (ΔΔCT) values were calculated based on the data generated with the QuantStudio Design & Analysis software v1.4.3 (Thermo Fisher Scientific).

## Results

### Clinical case reports

#### Patient 1

**Fig. 2:**
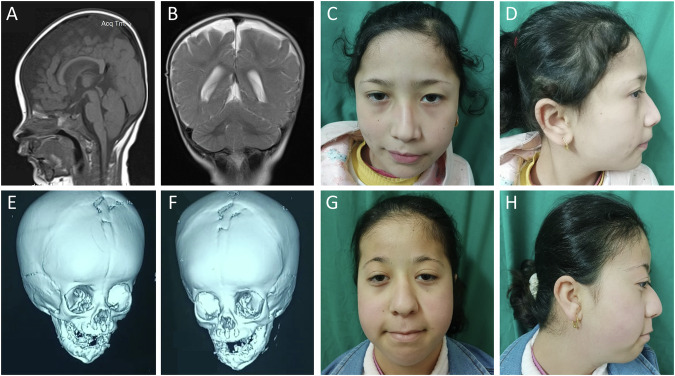
Craniofacial features of both patients, brain imaging of patient 1, and 3D CT skull of patient 2. Sagittal T_1_-weighted (**A**) and coronal T_2_-weighted (**B**) images of the brain of patient 1 at the age of 8 months showed no abnormalities. The cerebellar vermis and hemispheres had a normal appearance. **C** and **D** Patient 1 had high forehead, flat occiput, facial asymmetry, thin lateral and medial flaring of eyebrows, hypoplasia of the left upper eyelid, left epicanthic fold, upslanted palpebral fissures, deviated nasal septum with overhanging nasal tip, deep philtrum, downturned corners of the mouth, and multiple facial nevi. **E** and **F** 3D CT skull of patient 2 at the age of 2 months displayed premature fusion of the upper halves of coronal sutures and metopic sutures. **G** and **H** Patient 2 had high forehead, facial asymmetry, flat occiput, lateral thinning of the eyebrows, bilateral ptosis, downslanted palpebral fissures, depressed nasal bridge, deviated nasal septum, long philtrum, downturned corners of the mouth, thin upper lip, retrognathia, and multiple facial nevi

The 8-year-old girl was the youngest child of three siblings born to consanguineous healthy parents. She had a similarly affected sister (Patient 2) and a brother who died at the age of 8 months of unknown cause (Fig. 1C). Patient 1 was born by normal vaginal delivery at full term after an unremarkable pregnancy. Birth weight was 3 kg (−0.52 SD). Brain imaging at the age of 8 months revealed no abnormalities (Fig. 2A, B). She could walk alone at the age of 1 year and 2 months.

On clinical examination at the age of 7 years and 7 months, she showed distinct facial features (Fig. 2C, D). She had delayed speech and language development, mild intellectual disability (IQ 70), and an attention deficit hyperactivity disorder. Her height of 125 cm (+0.15 SD) and weight of 24 kg (−0.09 SD) were in the normal range, while her occipitofrontal circumference (OFC), at 48 cm (−2.79 SD), was disproportionally low. She had high-arched palate and short right 4th and 5th toes. Karyotyping revealed 46,XX.

#### Patient 2

The 13-year-old girl was patient 1’s sister and the first-born child of her parents. There were no complications during pregnancy and she was born by normal vaginal delivery at full term. Her birth weight was 2.5 kg (-1.73 SD). A 3D CT scan of her skull at 2 months of age revealed premature fusion of the upper halves of coronal sutures and metopic sutures (Fig. 2E, F). She had mitral valve prolapse and cleft palate that was surgically repaired at the age of 2 years; craniosynostosis was repaired at the age of 2 years and 6 months. At the age of 4 years, audiogram revealed bilateral conductive hearing loss due to repeated otitis media assessed by tympanometry. She could walk at the age of 1 year.

On clinical examination at the age of 13 years, she exhibited dysmorphic facial features (Fig. 2G, H). She had a low posterior hairline, prominent interdigital folds, bilateral hallux valgus with 2^nd^ toe overlapping the hallux, and hypoplastic toenails. Her growth parameters were within the normal range except OFC: height was 145 cm (−1.53 SD), weight was 35 kg (−1.5 SD), and OFC was 47 cm (−3.0 SD). Her speech and language development was delayed and she had moderate intellectual disability (IQ 36). Karyotyping revealed 46,XX.

### Genetic findings

WES in one sibling (patient 1) identified the homozygous *VLDLR* nonsense variant NM_003383.5:c.376C > T; p.(Gln126*) in exon 4, whose presence was confirmed in her sister (patient 2) in the homozygous state and in her parents in the heterozygous state (Fig. 1D). The single nucleotide variant has been reported in one heterozygous carrier with a worldwide allele frequency of 0.0000012 in the gnomAD database v4.0.0 [10]. As the majority of previously reported *VLDLR* pathogenic variants are loss-of-function variants [2], homozygosity of the novel *VLDLR* stop-gain variant likely underlies the neurodevelopmental disorder in the two sisters.

### *VLDLR* transcript analyses

**Fig. 3:**
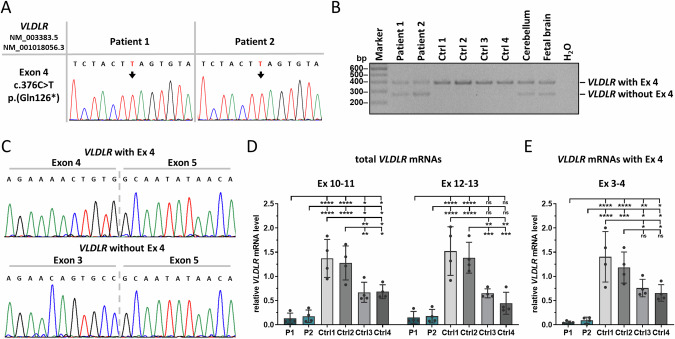
Qualitative and quantitative *VLDLR* transcript analysis in patient-derived fibroblasts. A Sequence traces showing *VLDLR* mutant mRNAs with the nonsense variant c.376C > T in fibroblasts from patients 1 and 2. An arrow points to the nucleotide change. **B** Agarose gel showing RT-PCR amplicons of *VLDLR* transcripts with primers located in exons 3 and 5 using cDNA derived from patient- and control-fibroblasts, human cerebellum, and fetal brain. The lower band of 272 bp represents *VLDLR* transcripts without exon 4 and the upper band (395 bp) transcripts with exon 4. **C** Sequence traces of colony PCR products from cloned RT-PCR amplicons of patient 1. The upper sequence shows *VLDLR* transcripts with exon 4 and the lower sequence *VLDLR* mRNAs without exon 4. Quantification of total *VLDLR* mRNA levels with two primer combinations (exons 10-11 and 12-13) (**D**) and of *VLDLR* exon 4-containing mRNA levels with primers in exons 3 and 4 (**E**) by RT-qPCR. *GAPDH* mRNA was used as an internal control; the amount of target mRNA relative to *GAPDH* mRNA is presented. The mean ± SD of four independent experiments, each performed in triplicate, is shown. One-way ANOVA with Dunnett’s correction was used for statistical analysis: *p ≤ 0.05; **p ≤ 0.01; ***p ≤ 0.001; ****p ≤ 0.0001; Ctrl control, Ex exon, ns not significant, P patient

To study the effects of the variant c.376C > T on the expression of *VLDLR* mRNAs, we used fibroblasts from patients 1 and 2 and confirmed the homozygous *VLDLR* nonsense variant c.376C > T; p.(Gln126*) in fibroblast DNA of both sisters (Supplementary Fig. S1). By using cDNA derived from fibroblasts of both patients, we generated RT-PCR amplicons with a forward primer spanning the *VLDLR* exon 3-exon 4 junction and a reverse primer in exon 5 to amplify *VLDLR* transcripts containing exon 4. Amplicon sequencing revealed the c.376 C > T variant in *VLDLR* transcripts of patient cells (Fig. 3A). To obtain alternatively spliced *VLDLR* transcripts, we used primers located in exons 3 and 5 and performed RT-PCR on cDNA from patient and control fibroblasts, human cerebellum, and human fetal brain. We generated a 395-bp RT-PCR product from all cDNA samples, while a 272-bp RT-PCR product was only amplified from cDNA of patient fibroblasts, human cerebellum, and fetal brain, but not from control fibroblasts (Fig. 3B). We cloned RT-PCR amplicons from both patients and cerebellum, performed colony PCR, and Sanger-sequenced colony PCR products. The larger amplicons represented *VLDLR* mRNAs with exon 4, while transcripts representing the smaller amplicons lack exon 4 (Fig. 3C**and** Supplementary Fig. S2). Importantly, *VLDLR* transcripts with exon 4 of both patients harbor the variant c.376C > T (Supplementary Fig. S2A, B). We investigated about 60 single *E. coli* colonies for each patient and identified 77.6% of colonies without exon 4 and 22.4% with exon 4 for patient 1 (Supplementary Fig. S2A) and 67.8% without exon 4 and 32.2% with exon 4 for patient 2 (Supplementary Fig. S2B). Together, the data show that *VLDLR* transcripts with exon 4 are predominantly expressed in human fibroblasts, while mRNAs with and without exon 4 can be detected in fibroblasts from both patients as well as in human cerebellum and fetal brain.

We determined total *VLDLR* transcript levels in patient and control fibroblasts by RT-qPCR. We used two primer pairs (in exons 10-11 and 12-13) located outside the alternatively spliced exons to amplify all four *VLDLR* mRNA species (Fig. 1A). We detected highly variable *VLDLR* mRNA levels in the four control fibroblast lines (Fig. 3D). Notably, *VLDLR* transcript levels were statistically significantly higher in fibroblasts of female controls 1 and 2 compared to male controls 3 and 4 (Fig. 3D). Higher liver *VLDLR* mRNA levels in young women compared to age-matched men have recently been reported in a preprint article (https://www.biorxiv.org/content/10.1101/2023.10.07.561324v3) that is in line with the sexual dimorphism of lipid metabolism in humans [11]. Total *VLDLR* mRNA levels were significantly reduced to 10-11% and 12-14% (for both primer pairs) in fibroblasts of patient 1 and patient 2, respectively, compared to the two female control cell lines 1 and 2 (Fig. 3D). Compared to the two male control cell lines 3 and 4, total *VLDLR* mRNA levels in patient 1 and patient 2 were significantly reduced to ~20% and ~26%, respectively, with primers in exons 10-11. For the second primer pair in exons 12-13, we detected a statistically non-significant reduction to 23-34% (*p* values of 0.072 and 0.43) and to 27-40% (*p* values of 0.095 and 0.524) in patient 1 and patient 2 cells, respectively, compared to the two male control cell lines 3 and 4 (Fig. 3D). To specifically determine the relative amount of *VLDLR* transcripts containing exon 4, we used primers in exons 3 and 4 in RT-qPCR experiments. In fibroblasts of patients 1 and 2, *VLDLR* transcripts with exon 4 were significantly reduced to 4-8% compared to the two female controls and to 7-14% compared to the two male controls (Fig. 3E). Together, our data show a reduction in total *VLDLR* mRNA levels, including all four transcript variants, and in exon 4-containing *VLDLR* mRNAs in fibroblasts of both patients. The results suggest that most of the *VLDLR* transcripts with exon 4 containing the premature stop codon are likely degraded by nonsense-mediated mRNA decay in patient-derived fibroblasts.

## Discussion

We here report on a neurodevelopmental disorder in two sisters with a homozygous nonsense variant c.376C > T; p.(Gln126*) in the alternatively spliced exon 4 of *VLDLR*. The phenotype in both siblings was less severe than previously reported for the *VLDLR* cerebellar hypoplasia [2]. Both siblings shared specific facial features, microcephaly, and mild to moderate ID. They did not show typical features of *VLDLR* cerebellar hypoplasia, such as truncal ataxia, dysarthria, and seizures. Brain imaging did not reveal the characteristic malformation pattern, including pontine and cerebellar hypoplasia, lack of vermis foliation, and frontotemporal pachygyria [2]. A 12-year-old patient with features of the *VLDLR* cerebellar hypoplasia that are at the mild end of the spectrum and a homozygous missense variant c.1256G > A; p.(Cys419Tyr) (Fig. 1A) has been reported. These data suggest that biallelic *VLDLR* missense variants can have a relatively mild impact on VLDLR protein function [12]. The two affected siblings shared several dysmorphic features (Fig. 2C, D, G, H). Although one patient with *VLDLR* cerebellar hypoplasia and high palate and triangular face has been reported [13], facial dysmorphism in this patient and the sisters described here may be related to other genes [2].

Pathogenic *VLDLR* variants in patients with the typical cerebellar hypoplasia phenotype affect exons that are present in all four transcript variants (Fig. 1A). The c.376C > T; p.(Gln126*) variant identified in the two sisters reported here is located in exon 4 that is absent in two of four *VLDLR* mRNAs (Fig. 1A). We hypothesized that expression of the two alternatively spliced *VLDLR* transcript variants lacking exon 4 may have partially compensated for the absence of the other two *VLDLR* mRNAs in certain cells of the brain. Alternative splicing of *VLDLR* exons has been reported in mammalians [5–7, 14], with exon 4-containing and -lacking transcripts expressed in human brain [7]. *VLDLR* transcripts lacking exons 4 and 16 are expressed in human cerebellum. In neuronal and astrocyte cultures from mouse cerebral cortex and cerebellum, exon 4-lacking *Vldlr* mRNAs were expressed in neurons and rarely in astrocytes [6]. The regulation of alternative splicing of human *VLDLR* pre-mRNAs is apparently complex and developmentally regulated [6, 14, 15], suggesting the production of cell-type specific VLDLR isoforms.

VLDLR binds many different ligands, including apolipoprotein E and Reelin in the brain [3, 4]. VLDLR isoforms I to IV have different ligand binding properties. For example, VLDLR-III lacking the third LA repeat has a higher binding capacity of apolipoprotein E than VLDLR-I and -II [6]. The VLDLR ligand Reelin is an extracellular matrix protein which is important for the formation of the six-layered neocortex [16]. Reelin binds the first LA repeat of VLDLR [17] and the Reelin-VLDLR complex is required to terminate neuronal migration [18]. The brain platelet-activating factor acetylhydrolase 1b (Pafah1b) complex, including Lis1 (*Pafah1b1)*, is involved in Reelin signaling and neuronal migration [19]. Two catalytic subunits of the Pafah1b complex specifically bind to VLDLR [20]. Biallelic variants in *RELN* encoding Reelin *cause l*issencephaly and hypoplasia or dysplasia of the cerebellum and hippocampus [21] and heterozygous *PAFAH1B1* variants are associated with lissencephaly [22]. The absence of brain malformations in the two siblings reported here suggests that neuronal VLDLR isoforms lacking the third LA repeat are able to bind Reelin and the Pafah1b complex to regulate brain development and neuronal migration. However, VLDLR-III and -IV are unlikely to fully compensate for the lack of VLDLR-I and -II in the brain, and binding of VLDLR-III and -IV to specific ligands important for brain development could be impaired. A search of the BioGRID database identified SYT1 (synaptotagmin I) as a potential binding partner of VLDLR [23]. Heterozygous *SYT1* variants cause a neurodevelopmental disorder with global developmental delay and other abnormalities, but without seizures and brain anomalies [24]. Thus, impaired binding of VLDLR-III and -IV to SYT1 may be one scenario that could underlie the neurological phenotype in the patients reported here.

## Supplementary information

Supplemental Material

ICMJE DISCLOSURE FORM

## Data Availability

The data that support the findings of this study are available from the corresponding author upon reasonable request.
