# Supplementary material for: A homozygous nonsense variant in the alternatively spliced VLDLR exon 4 causes a neurodevelopmental disorder without features of VLDLR cerebellar hypoplasia
Source: J Hum Genet. 2024 Jul 31;69(12):623–8. doi: 10.1038/s10038-024-01279-w (PMC11599036; doi:10.1038/s10038-024-01279-w)
Supplement: Supplementary file 1 — Supplemental Material [file 10038_2024_1279_MOESM1_ESM.docx]

**Supplementary Material**

**Article**

**A homozygous nonsense variant in the alternatively spliced *VLDLR* exon 4 causes a neurodevelopmental disorder without features of *VLDLR* cerebellar hypoplasia**

Tess Holling,^1^ Ibrahim M. Abdelrazek,^2^ Ghada M. Elhady,^2^ Marwa Abd Elmaksoud,^3^ Seung Woo Ryu,^4^ Ebtesam Abdalla,^2^ Kerstin Kutsche^1^

^1^Institute of Human Genetics, University Medical Center Hamburg-Eppendorf, Hamburg, Germany

^2^Department of Human Genetics, Medical Research Institute, Alexandria University, Alexandria, Egypt

^3^Neurology Unit, Pediatric Department, Faculty of Medicine, Alexandria University, Egypt

^4^3billion Inc., Seoul, South Korea

Ibrahim M. Abdelrazek is co-first author.

Correspondence to:

Kerstin Kutsche, PhD

Institute of Human Genetics

University Medical Center Hamburg-Eppendorf

Martinistraße 52

20246 Hamburg

Germany

email: [kkutsche@uke.de](mailto:kkutsche@uke.de)

**
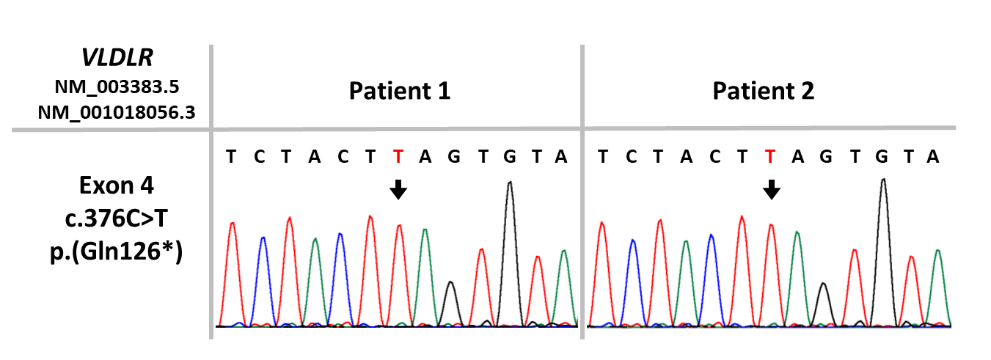
**

**Figure S1: *VLDLR* variant validation in patient-derived fibroblasts.**

Partial sequence traces showing the *VLDLR* nonsense variant c.376C>T; p.(Gln126*) in DNA samples derived from fibroblasts of patients 1 and 2 in the homozygous state. An arrow points to the nucleotide change.

**
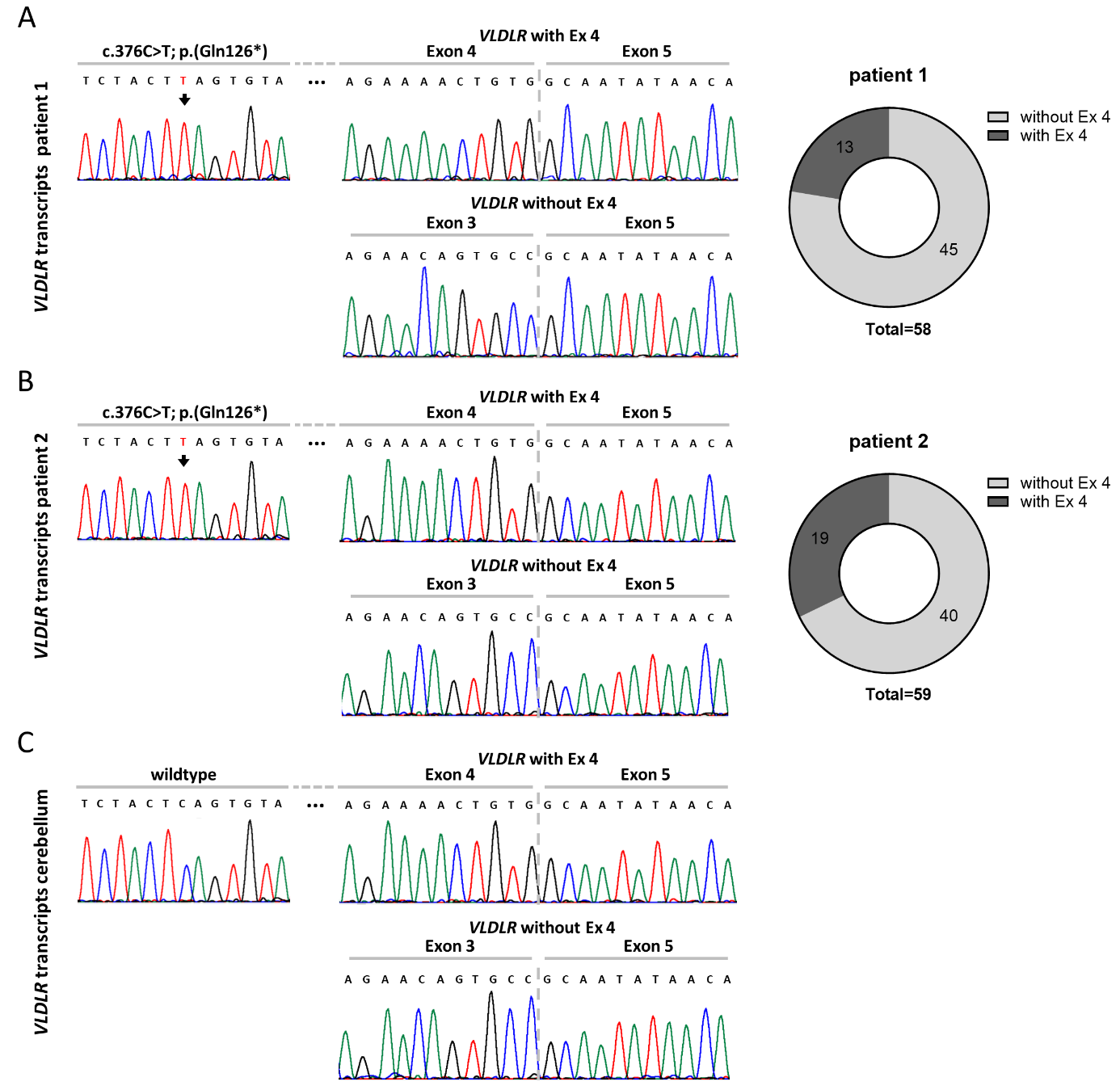
**

**Figure S2: *VLDLR* transcript variants identified by sequencing of single colony PCR products.**

Partial sequence electropherograms of single colony PCR products from cloned RT-PCR amplicons (see Figure 3B) of patient 1 (**A**), patient 2 (**B**) and human cerebellum (**C**). In the upper panels, sequence traces show *VLDLR* transcripts with exon 4 (NM_003383.5 and NM_001018056.3). An arrow points to the c.376C>T change. In the lower panels, sequences of *VLDLR* mRNAs without exon 4 are shown (NM_001322225.2 and NM_001322226.2). Pie charts on the right (**A** and **B**) show the number of transcripts with and without exon 4 detected in 58 *E. coli* colonies for patient 1 (**A**) and in 59 *E. coli* colonies for patient 2 (**B**). Ex: exon.

**Table S1: Published *VLDLR* variants, associated phenotypes with MRI findings, and number of patients in the publications.**

| ***VLDLR* variant (NM_003383.5)** | | | **Phenotype** | | | **Reference** |
| --- | --- | --- | --- | --- | --- | --- |
| **Nucleotide change** | **Change at  protein level** | **Exon** | **Phenotype listed in Human Gene Mutation Database (HGMD)** | **Number of patients** | **MRI findings** |  |
| c.22dupG | p.(Ala8Glyfs*27) | 1 | Cerebellar hypoplasia | 2 | Hypoplasia of cerebellar vermis and hemispheres, minimal atrophy of the brainstem, thickening of the bilateral commissural fibers | ^1^ |
| c.150_151delGA | p.(Trp50*) | 2 | Cerebellar ataxia, mental retardation and dysequilibrium syndrome | 1^a^ | Neocortical defects, pons hypoplasia and profound cerebellar involvement with the absence of the inferior vermis and abnormal foliation | ^2^ |
| c.154T>C | p.(Cys52Arg) | 2 | Intellectual disability/developmental delay | 1 | Hypoplasia of cerebellar vermis, normal cerebral gyration | ^3^ |
| c.658_743del | p.(Asp220Trpfs*6) | 5 | Dysequilibrium syndrome | 10 | Cerebellar hypoplasia, decrease in the cortical sulci | ^4^ |
| c.767G>A | p.(Trp256*) | 5 | Cerebellar hypoplasia and mental retardation with or without quadrupedal locomotion 1 | 7 | No brain MRI available | ^5^ |
| c.769C>T | p.(Arg257*) | 5 | Cerebellar hypoplasia and quadrupedal locomotion | 10 | Inferior cerebellar and vermial hypoplasia, with the inferior vermial portion being completely absent. Normal corpus callosum, moderate simplification of the cerebral cortical gyri, particularly small brainstem and pons | ^6^ |
| c.820+1G>A | – | Intron 6 | Cerebellar ataxia, mental retardation and dysequilibrium syndrome | 2^b^ | Neocortical defects, pons hypoplasia and profound cerebellar involvement with the absence of the inferior vermis and abnormal foliation | ^2^ |
| c.835C>T | p.(Arg279*) | 6 | Pontocerebellar hypoplasia, cortical dysplasia, mental retardation and bipedal gait | 1 | Bilateral diffuse cerebral cortical thickening, decreased gyration in accordance with pachygyria, cerebellar and pontine hypoplasia | ^7^ |
| c.901C>T | p.(Arg301*) | 6 | Cerebellar ataxia, mental retardation and dysequilibrium syndrome | 2^b^ | Neocortical defects, pons hypoplasia and profound cerebellar involvement with the absence of the inferior vermis and abnormal foliation | ^2^ |
| c.1249_1255delTACAAGT | p.(Tyr417Valfs*19) | 9 | Cerebellar ataxia with intellectual disability | 2 | Pontocerebellar hypoplasia | ^8^ |
|  |  |  |  | 2 | Diffuse pachygyria of the cerebrum, hypoplasia of the pons and cerebellum | ^7^ |
| c.1256G>A | p.(Cys419Tyr) | 9 | Dysequilibrium syndrome | 1 | Severe cerebellar hypoplasia with absent folia of the vermis, marked pontine hypoplasia, simplified gyral pattern | ^9^ |
| c.1342C>T | p.(Arg448*) | 10 | Dysequilibrium syndrome | 8 | No brain MRI available | ^10^ |
|  |  |  |  | 3 | No brain MRI available | ^11^ |
| c.1459G>T | p.(Asp487Tyr) | 10 | Lissencephaly with cerebellar hypoplasia | 1 | Cerebellar hypoplasia, particularly affecting the inferior hemispheres and vermis as a whole, small pons, mild to moderate cortical thickening (pachygyria), simplification of the gyral architectural folding | ^12^ |
|  |  |  |  | 2 | Pachygyria and cerebellar hemisphere hypoplasia | ^13^ |
| c.1561G>C | p.(Asp521His) | 11 | Dysequilibrium syndrome | 1^c^ | Hypoplasia of the inferior vermis and cerebellar hemispheres, small pons, mild simplification of the sulcation pattern, slightly thickened cerebral cortex, lack of clear anteroposterior gradient | ^14^ |
| c.1586G>A | p.(Trp529*) | 11 | Cerebellar ataxia, mental retardation and dysequilibrium syndrome | 1 | No brain MRI available | ^15^ |
| c.1711dupT | p.(Tyr571Leufs*7) | 12 | Dysequilibrium syndrome | 1^c^ | Hypoplasia of the inferior vermis and cerebellar hemispheres, small pons mild simplification of the sulcation pattern, slightly thickened cerebral cortex, lack of clear anteroposterior gradient | ^14^ |
| c.1724G>A | p.(Trp575*) | 12 | Cerebellar ataxia, mental retardation and dysequilibrium syndrome | 1 | Neocortical defects, pons hypoplasia and profound cerebellar involvement with the absence of the inferior vermis and abnormal foliation | ^2^ |
| c.1961A>G | p.(Glu654Gly) | 13 | Cerebellar ataxia, mental retardation and dysequilibrium syndrome | 1^a^ | Neocortical defects, pons hypoplasia and profound cerebellar involvement with the absence of the inferior vermis and abnormal foliation | ^2^ |
| c.2117G>T | p.(Cys706Phe) | 15 | Dysequilibrium syndrome | 5 | Absent inferior vermis and hypoplastic inferior-cerebellum, neocortical pachygyria and mega cisterna magna, typical square-shaped midbrain-hindbrain junction morphology | ^16^ |
| c.2240G>T | p.(Arg747Leu) | 15 | Cerebellar hypoplasia and quadrupedal locomotion | 4 | Typical manifestations | ^17^ |
| c.2248C>T | p.(Gln750*) | 15 | Ataxia with vitamin E deficiency | 2 | Pachygyria, profound cerebellar volume loss, absent foliation, “tigroid” appearance to the subcortical white matter | ^18^ |
| c.2339delT | p.(Ile780Thrfs*3) | 17 | Cerebellar hypoplasia and quadrupedal locomotion | 3 | Cerebral cortical simplification, inferior cerebellar and vermial hypoplasia | ^6^ |
| 199 kb deletion between *D9S129* and *D9S1871* | | all | Dysequilibrium syndrome | 10 | Small cerebellum in a fluid-filled but normal-sized posterior fossa, small pons, simplified cerebral cortex | ^19^ |
| chr9:2,621,092-2,630,805del including exon 1 | | 1 | Pachygyria & pontocerebellar atrophy | 2 | Diffuse pachygyria of the cerebrum, hypoplasia of the cerebellar vermis | ^20^ |
| 32 kb deletion including exon 1 | | 1 | Intellectual disability and severe ataxia | 2 | No brain MRI available | ^21^ |

^a, b, c^: compound heterozygous variants in the indicated patient(s).

**Table S2: Sequence of oligonucleotides used in this work.**

| **Primer sequences for *VLDLR* variant validation** | | | |
| --- | --- | --- | --- |
| **Template** | **Intron** | **Direction** | **Sequence (5’ → 3’)** |
| genomic DNA | 3 | forward | CCT TGA AGG AGT GAC GTG GT |
|  | 4 | reverse | TGA AGG ACA AGT AAT TGA GCA GA |
| **Primer sequences for qualitative *VLDLR* transcript analysis** | | | |
| **Template** | **Exon** | **Direction** | **Sequence (5’ → 3’)** |
| cDNA  (fibroblasts) | 3 | forward | TGA CTG CGA AGA TGG TTC AG |
|  | 3/4 | forward | CAG TGC CAT ATG AGA ACA TGC C |
|  | 5 | reverse | CCA GGG ACT CAT CAG ATT GG |
| **Primer sequences for quantitative *VLDLR* transcript analysis** | | | |
| **Template** | **Exon** | **Direction** | **Sequence (5’ → 3’)** |
| cDNA  (fibroblasts) | 3 | forward | GTG ATG GAG ATC CTG ACT GC |
|  | 4 | reverse | TTT CTT CAT CTT CTC CAC TGT CAC |
|  | 10 | forward | CTG ACA TTG CTG CCC AGA AAC |
|  | 11 | reverse | TCT TAG AAG CCG CAT CAG TCC |
|  | 12 | forward | GCA GGA ATG AAT GGA TTC GAT AGA C |
|  | 13 | reverse | GGA TGA GCT AGG AAC TCC AGA GAC |

**References:**

1. Yuce Kahraman C, Ercoskun P, Yakar O, Tatar A. A novel genomic variant in two siblings with very low-density lipoprotein receptor-associated cerebellar hypoplasia. Clin Dysmorphol. 2022;31:98-100.

2. Valence S, Garel C, Barth M, Toutain A, Paris C, Amsallem D, et al. RELN and VLDLR mutations underlie two distinguishable clinico-radiological phenotypes. Clin Genet. 2016;90:545-49.

3. Giorgio E, Ciolfi A, Biamino E, Caputo V, Di Gregorio E, Belligni EF, et al. Whole exome sequencing is necessary to clarify ID/DD cases with de novo copy number variants of uncertain significance: Two proof-of-concept examples. Am J Med Genet A. 2016;170:1772-9.

4. Wali GM, Wali G. Broadening the Clinical Spectrum of Very Low Density Lipoprotein Receptor Associated Dysequilibrium Syndrome. Mov Disord Clin Pract. 2021;8:619-23.

5. Gorukmez O, Gorukmez O, Topak A. Clinical exome sequencing findings in 1589 patients. American Journal of Medical Genetics Part A. 2023;191:1557-64.

6. Ozcelik T, Akarsu N, Uz E, Caglayan S, Gulsuner S, Onat OE, et al. Mutations in the very low-density lipoprotein receptor <i>VLDLR</i> cause cerebellar hypoplasia and quadrupedal locomotion in humans. Proceedings of the National Academy of Sciences. 2008;105:4232-36.

7. Sonmez FM, Gleeson JG, Celep F, Kul S. The very low density lipoprotein receptor-associated pontocerebellar hypoplasia and dysmorphic features in three Turkish patients. J Child Neurol. 2013;28:379-83.

8. Dixon-Salazar TJ, Silhavy JL, Udpa N, Schroth J, Bielas S, Schaffer AE, et al. Exome sequencing can improve diagnosis and alter patient management. Sci Transl Med. 2012;4:138ra78.

9. Micalizzi A, Moroni I, Ginevrino M, Biagini T, Mazza T, Romani M, et al. Very mild features of dysequilibrium syndrome associated with a novel VLDLR missense mutation. Neurogenetics. 2016;17:191-5.

10. Moheb LA, Tzschach A, Garshasbi M, Kahrizi K, Darvish H, Heshmati Y, et al. Identification of a nonsense mutation in the very low-density lipoprotein receptor gene (VLDLR) in an Iranian family with dysequilibrium syndrome. European Journal of Human Genetics. 2008;16:270-73.

11. Hu H, Kahrizi K, Musante L, Fattahi Z, Herwig R, Hosseini M, et al. Genetics of intellectual disability in consanguineous families. Mol Psychiatry. 2019;24:1027-39.

12. Azmanov DN, Chamova T, Tankard R, Gelev V, Bynevelt M, Florez L, et al. Challenges of diagnostic exome sequencing in an inbred founder population. Mol Genet Genomic Med. 2013;1:71-6.

13. Elmas M, Yıldız H, Erdoğan M, Gogus B, Avcı K, Solak M. Comparison of clinical parameters with whole exome sequencing analysis results of autosomal recessive patients; a center experience. Mol Biol Rep. 2019;46:287-99.

14. Boycott KM, Bonnemann C, Herz J, Neuert S, Beaulieu C, Scott JN, et al. Mutations in VLDLR as a cause for autosomal recessive cerebellar ataxia with mental retardation (dysequilibrium syndrome). J Child Neurol. 2009;24:1310-5.

15. Capalbo A, Valero RA, Jimenez-Almazan J, Pardo PM, Fabiani M, Jiménez D, et al. Optimizing clinical exome design and parallel gene-testing for recessive genetic conditions in preconception carrier screening: Translational research genomic data from 14,125 exomes. PLOS Genetics. 2019;15:e1008409.

16. Ali BR, Silhavy JL, Gleeson MJ, Gleeson JG, Al-Gazali L. A missense founder mutation in VLDLR is associated with Dysequilibrium Syndrome without quadrupedal locomotion. BMC Med Genet. 2012;13:80.

17. Al-Gazali L, Ali BR. Mutations of a country: a mutation review of single gene disorders in the United Arab Emirates (UAE). Hum Mutat. 2010;31:505-20.

18. Kruer MC, Jepperson TN, Weimer JM, Mroch A, Davis-Keppen L, Crotwell P, et al. Mutations in VLDLR associated with ataxia with secondary vitamin E deficiency. Mov Disord. 2013;28:1904-5.

19. Boycott KM, Flavelle S, Bureau A, Glass HC, Fujiwara TM, Wirrell E, et al. Homozygous deletion of the very low density lipoprotein receptor gene causes autosomal recessive cerebellar hypoplasia with cerebral gyral simplification. Am J Hum Genet. 2005;77:477-83.

20. Kolb LE, Arlier Z, Yalcinkaya C, Ozturk AK, Moliterno JA, Erturk O, et al. Novel VLDLR microdeletion identified in two Turkish siblings with pachygyria and pontocerebellar atrophy. Neurogenetics. 2010;11:319-25.

21. Makrythanasis P, Nelis M, Santoni FA, Guipponi M, Vannier A, Béna F, et al. Diagnostic exome sequencing to elucidate the genetic basis of likely recessive disorders in consanguineous families. Hum Mutat. 2014;35:1203-10.
